# PL3 Amidase, a Tailor-made Lysin Constructed by Domain Shuﬄing with Potent Killing Activity against Pneumococci and Related Species

**DOI:** 10.3389/fmicb.2016.01156

**Published:** 2016-07-28

**Authors:** Blas Blázquez, Alba Fresco-Taboada, Manuel Iglesias-Bexiga, Margarita Menéndez, Pedro García

**Affiliations:** ^1^Departamento de Microbiología Molecular y Biología de las Infecciones, Centro de Investigaciones Biológicas, Consejo Superior de Investigaciones CientíficasMadrid, Spain; ^2^Departamento de Química-Física Biológica, Instituto Química-Física Rocasolano, Consejo Superior de Investigaciones CientíficasMadrid, Spain; ^3^CIBER de Enfermedades RespiratoriasMadrid, Spain

**Keywords:** lysin, pneumococcus, phage therapy, chimeric protein, *Streptococcus oralis*, *Streptococcus mitis*, *Streptococcus pseudopneumoniae*

## Abstract

The emergence and spread of antibiotic-resistant bacteria is pushing the need of alternative treatments. In this context, phage therapy is already a reality to successfully fight certain multiresistant bacteria. Among different phage gene products, murein hydrolases responsible of phage progeny liberation (also called lysins or endolysins) are weapons that target specific peptidoglycan bonds, leading to lysis and death of susceptible bacteria when added from the outside. In the pneumococcal system, all but one phage murein hydrolases reported to date share a choline-binding domain that recognizes cell walls containing choline residues in the (lipo)teichoic acids. Some purified pneumococcal or phage murein hydrolases, as well as several chimeric proteins combining natural catalytic and cell wall-binding domains (CBDs) have been used as effective antimicrobials. In this work we have constructed a novel chimeric *N*-acetylmuramoyl-L-alanine amidase (PL3) by fusing the catalytic domain of the Pal amidase (a phage-coded endolysin) to the CBD of the LytA amidase, the major pneumococcal autolysin. The physicochemical properties of PL3 and the bacteriolytic effect against several pneumococci (including 48 multiresistant representative strain) and related species, like *Streptococcus pseudopneumoniae, Streptococcus mitis*, and *Streptococcus oralis*, have been studied. Results have shown that low doses of PL3, in the range of 0.5–5 μg/ml, are enough to practically sterilize all choline-containing strains tested. Moreover, a single 20-μg dose of PL3 fully protected zebrafish embryos from infection by *S. pneumoniae* D39 strain. Importantly, PL3 keeps 95% enzymatic activity after 4 weeks at 37°C and can be lyophilized without losing activity, demonstrating a remarkable robustness. Such stability, together with a prominent efficacy against a narrow spectrum of human pathogens, confers to PL3 the characteristic to be an effective therapeutic. In addition, our results demonstrate that the structure/function-based domain shuﬄing approach is a successful method to construct tailor-made endolysins with higher bactericidal activities than their parental enzymes.

## Introduction

Discovery of penicillin and other antibiotics allowed effective treatment of infectious diseases, which provoked a tremendous impact on public health. However, it has been learned that sooner or later bacteria are capable of acquiring resistance to practically every known antibiotic. This resistance is readily transferred to other bacteria and, at the end, there is a continuous warfare between the ability of bacteria to resist any new antimicrobial and the armamentarium of new weapons to overcome treatment failures and kill the targeted bacteria. In this context, the use and abuse of antibiotics in the last years have led to a substantial rise of bacterial multiresistance and this worrying situation runs parallel with the scarcity of new antimicrobials in the pharmaceutical pipeline (Spellberg et al., 2015).

Among the main human pathogens, *Streptococcus pneumoniae* continues to be a major cause of morbidity and mortality worldwide —causing more deaths than any other infectious disease —being children younger than 5 years old, the elderly, and immunocompromised people the major groups at risk. Pneumococcal diseases range from mild infections, such as otitis media and sinusitis, to more severe diseases such as pneumonia (either invasive or not), septicemia, and meningitis. Despite the availability of vaccines and antibiotics, a recent report estimated that pneumococcus is still responsible for approximately 1.3 million deaths annually (Walker et al., 2013). For decades, the standard treatment of pneumococcal infections has been penicillin, to which this species was exquisitely sensitive. However, a widespread increase of pneumococci resistant to most antibiotics (except to vancomycin) has been progressively observed (Kim et al., 2016). The tendency on the emergence of multidrug resistance pathogens is an increasingly global economic and healthcare crisis, and this situation is pushing to find alternative approaches for combating such pathogens, *S. pneumoniae* being one of the more clear examples (Huttner et al., 2013).

Bacteriophage-encoded lytic enzymes (or endolysins) are murein hydrolases that selectively break different bonds of peptidoglycan, thereby enabling the release of progeny virions at the end of the infection cycle of the majority of double-stranded DNA bacteriophages. Purified endolysins, and bacterial autolysins as well, can be applied exogenously to brake the bacterial cell wall in an effective and selective way. This novel class of antibacterials, also known as enzybiotics, presents important advantages over classical antibiotics, e.g., narrow spectrum of susceptible bacteria, rapid killing of stationary and logarithmically growing bacteria, and low probability to bacterial resistance (Pastagia et al., 2013). In addition, lysins can also eliminate bacteria from mucous membranes and bacterial biofilms, which are major reservoirs and routes of infection (Pastagia et al., 2011; Díez-Martínez et al., 2015). Typically, lysins from Gram-positive bacteria and their bacteriophages consist of a two-domain structure, but some of them have multiple hydrolytic domains or distinct types of cell wall-binding domains (CBDs; Rigden et al., 2003; Nelson et al., 2012). The stringent range of activity is primarily linked to the specificity of binding of the CBDs (Hermoso et al., 2007; Gilmer et al., 2013). However, the net charge of the domains and the fine architecture of the bacterial envelope contribute as well (Low et al., 2011; Díez-Martínez et al., 2013). Besides, the intrinsic activity of the catalytic domain, the strength of attachment to the cell wall, and the overall protein structure determine the actual lysis rate.

In the last years, several reports of endolysins showing strong lethal activity against relevant Gram-positive pathogens have been published, including *Staphylococcus aureus* (Rashel et al., 2007; Gilmer et al., 2013), enterococci (Yoong et al., 2004), the spore formers *Bacillus* and *Clostridium* genera (Nakonieczna et al., 2015), and even some Gram-negative pathogens like *Acinetobacter baumannii* (Lood et al., 2015). Specifically in pneumococcus, endolysins like the Cpl-1 and Cpl-7 lysozymes and derived chimeras (Díez-Martínez et al., 2013, 2015), or the Pal *N*-acetylmuramoyl-L-alanine amidase (NAM-amidase; EC 3.5.1.28; Loeﬄer et al., 2001; Jado et al., 2003) have been proved to kill efficiently several strains *in vitro* and *in vivo*. Moreover, the bactericidal effect of the major pneumococcal autolysin, the LytA NAM-amidase, against encapsulated *S. pneumoniae* cells has also been demonstrated (Rodríguez-Cerrato et al., 2007; Díez-Martínez et al., 2013). Pal and LytA have unrelated catalytic domains belonging to *Amidase_5* and *Amidase_2* families, respectively, which are fused to homologous choline-binding domains (66% sequence identity) that anchor to the phosphocholine residues of pneumococcal (lipo)teichoic acids (Sheehan et al., 1997). Both choline-binding domains are made up of six sequence-conserved repeats and a C-terminal tail, where choline moieties bind at the interface of every two-consecutive repeats, as deduced by the elucidation of the crystallographic structures of full-length LytA and its isolated choline-binding domain (Fernández-Tornero et al., 2001; Li et al., 2015). Besides, LytA contains a non-canonical choline-binding site in the first repeat of the choline-binding domain (Mellroth et al., 2014; Li et al., 2015). Based on the previous structural and functional knowledge of both NAM-amidases (Varea et al., 2000 and references therein; Fernández-Tornero et al., 2001; Varea et al., 2004; Li et al., 2015), we have constructed a novel chimeric lysin, PL3, by shuﬄing the catalytic domain of Pal with the choline-binding domain of LytA. PL3 turned out to be a potent enzybiotic against pneumococci and other choline-containing Gram-positive pathogens, and its lethality against pneumococcal encapsulated and multiresistant isolates was higher than those of the parental enzymes. In addition, *in vitro* bactericidal activity of PL3 has also been confirmed *in vivo* using a zebrafish embryo infection model.

## Materials and Methods

### Bacterial Strains and Growth Conditions

Bacterial strains used in this study are listed in **Table 1**. Pneumococcal cultures were grown at 37°C without aeration in C medium supplemented with 0.08% (w/v) yeast extract (C + Y medium; Lacks and Hotchkiss, 1960). Other Gram-positive bacteria were grown in brain heart infusion broth (Becton, Dickinson and Company) at 37°C without shaking. *Escherichia coli* strains were grown in LB medium with aeration at 37°C, supplemented with kanamycin (Km; 50 μg/ml) when required.

**Table 1:** Bacterial strains and plasmids.

Strains or plasmids	Genotype or description^a^	Reference^b^
**Strains**		
*Escherichia coli* BL21(DE3)	F^-^, *ompT, hsdSB* (*r_B_*^-^*m_B_*^-^), *gal, dcm*, aaaaDE3 (harboring gene *1* of the RNA polymerase from the phage T7 under the *PlacUV5* promoter)	Sambrook and Russell, 2001
*Streptococcus pneumoniae*		
R6	Standard laboratory strain, non-encapsulated	Hoskins et al., 2001
P046	R6 but *lytA, lytC*	Moscoso et al., 2006
D39	Serotype 2	Lanie et al., 2007
P007	R6 derivative, serotype 3	Domenech et al., 2009
P008	R6 derivative, serotype 4	Moscoso et al., 2006
48	Serotype 23F; penicillin MIC = 16 mg/ml; erythromycin MIC > 128 mg/ml; ciprofloxacin MIC = 1 mg/ml; levofloxacin MIC = 1 mg/ml; chloramphenicol MIC = 4 mg/ml; tetracycline MIC > 64 mg/ml	Soriano et al., 2008
*Streptococcus mitis*^T^	Type strain	NCTC 12261
*Streptococcus mitis* SK598	Biovar 1 strain with ethanolamine-containing C-polysaccharide	Bergström et al., 2003
*Streptococcus oralis*^T^	Type strain	NCTC 11427
*Streptococcus pseudopneumoniae*^T^	Type strain	ATCC BAA-960
**Plasmids**		
pET29a(+)	Expression vector; Km^R^	Novagen
pET29-PL3	pET29a(+), *pl3*; Km^R^	This study
pMSP11	Recombinant plasmid with *pal*	Sheehan et al., 1997
pMMN1	Recombinant plasmid with *lytA*	Moscoso et al., 2011

*^a^MIC, minimal inhibitory concentration.*

*^b^NCTC, National Collection of Type Cultures; ATCC, American Type Culture Collection.*

### Cloning, Expression, and Purification of PL3

The Pal encoding region was PCR amplified with primers 5’Nde_Pal (GGAGGGAAGACATATGGGAGTCGATATTGAAAAAGG, where the NdeI site is underlined) and 3′GYM_Pal (CGGTCTGCAAGCATGTAGCCTTGGTCGTCAAAG), using pMSP11 as template (Sheehan et al., 1997). The LytA encoding region was PCR amplified with primers 5′LA_LytA (CTTTGACGACCAAGGCTACATGCTTGCAGACCG) and 3′BamHI_LytA (CGCGGATCCTTATTTTACTGTAATCAAGCCATCTG, where the BamHI site is underlined) using pMMN1 as template (Moscoso et al., 2011). The resulting PCR products were used for a third PCR round to amplify the chimeric PL3 encoding gene, which was digested with NdeI and BamHI and cloned into pET29a(+) previously treated with the same enzymes. The resulting recombinant plasmid, pET29-PL3, was sequenced to ensure the accuracy of the insert and transformed into *E. coli* BL21(DE3). For overproduction of PL3, transformed cells were incubated to an optical density at 600 nm (OD_600_) of 0.6. Isopropyl-β-D-galactopyranoside (0.1 mM) was then added, and incubation continued for 4 h at 37°C. Cells were harvested by centrifugation (10000 × *g*, 5 min), resuspended in 20 mM sodium phosphate buffer (hereafter, PB), 0.5 M NaCl, pH 6.9, disrupted in a French pressure cell and ultracentrifuged (50000 × *g*, 45 min) to remove cell debris. Streptomycin sulfate (Sigma; 2%, w/v) was added to the protein extract and the mixture was incubated for 15 min at 4°C with slow stirring to facilitate DNA precipitation. The insoluble fraction was removed by ultracentrifugation (50000 × *g*, 45 min) at 4°C, and PL3 was purified from the supernatant by affinity chromatography using DEAE-cellulose (Sanz and García, 1990) followed by size exclusion chromatography on dextran-agarose (HiLoad 16/60 Superdex 200 PG column, GE Healthcare) to remove large protein aggregates. Briefly, PL3 fractions eluted from the affinity column were pooled, dialyzed against PB, pH 6.8, and subjected to gel filtration using the same buffer at a flow rate of 0.8 ml/min. The purity and state of the PL3 samples were checked by 12% SDS-PAGE and mass spectrometry (MALDI-TOF). Large-aggregate free fractions of PL3 were pooled, dialyzed against PB containing 100 mM NaCl, 25 mM choline, pH 6.8, and stored at -20°C. Before use, the protein was dialyzed against PB, pH 6.8, supplemented with 1 mM β-mercaptoethanol or 10 mM 1,4-dithiothreitol (DTT) when required. PL3 concentration was determined spectrophotometrically using the theoretical molar absorption coefficient at 280 nm (133855 M^-1^ cm^-1^, considering cysteine residues in the oxidized state).

### Mass Spectrometry

Purified PL3 samples were analyzed by MALDI-TOF in a Voyager DEPRO (Applied Biosystems), as described elsewhere (Moreno et al., 2008). A grid voltage of 89%, a 0.25 ion guide wire voltage, and a delay time of 400 ns in the linear positive-ion mode were used. External calibration was performed with carbonic anhydrase (29024 Da) and enolase (46672 Da) from Sigma, covering an *m/z* range of 10000–80000 units.

### Circular Dichroism (CD)

Circular dichroism spectra were recorded at 20°C with a J-810 spectropolarimeter (Jasco Corporation) equipped with a Peltier-type cell holder, using 1-mm (far-UV) or 10-mm (near-UV) path-length quartz cells and protein concentrations of 0.13 and 0.44 mg/ml, respectively (Bustamante et al., 2010). The buffer contribution was subtracted from the raw data and the corrected spectra were converted to mean residue ellipticities (Θ) using an average molecular mass per residue of 104.5. Spectra acquisition and analysis were carried out with the Spectra Manager software.

PL3 titration with choline was performed by measuring the CD spectra at varying choline concentrations and plotting the ellipticity variation at selected wavelengths as a function of choline concentration. To minimize errors, titrations were carried out by serial addition of small volumes of concentrated choline stocks to the same protein sample (less than 10% total volume increase). Choline stock concentrations were measured by differential refractometry (Usobiaga et al., 1996).

### Analytical Ultracentrifugation

Sedimentation velocity experiments were carried out in an Optima XL-A analytical ultracentrifuge (Beckman Coulter) at 20°C. Measurements were performed in PB, pH 6.8, at 45000 rpm using cells with double sector Epon-charcoal centerpieces (0.11 mg/ml PL3). Differential sedimentation coefficients were calculated by least-squares boundary modeling of the experimental data, and normalized to values in water at 20°C (*s_20,w_*), with the program SEDFIT (Brown and Schuck, 2006). The fractional friction coefficients (*f/f*_0_) and the Stokes radii (*R*_s_), related to the protein hydrodynamic shape, were calculated from the molecular masses and *s_20,w_* values using the partial specific volumes and hydration coefficients estimated from the amino acid sequence with the SEDNTERP program (Laue et al., 1992).

### Differential Scanning Calorimetry (DSC)

Differential scanning calorimetry measurements were performed at a heating rate of 60°C/h in a VP-DSC microcalorimeter (Microcal, Inc.), under an extra constant pressure of 1.8 atm, at 0.4 mg/ml PL3. Origin DSC software (Microcal) was used for data acquisition and analysis. The excess heat capacity function was obtained after subtraction of the buffer-buffer base line registered before each protein scan. Reheating of previously scanned samples showed that thermal denaturation of PL3 was totally or partially irreversible, depending on the buffer pH.

### *In Vitro* Cell Wall Activity Assay

Purified PL3 was checked for *in vitro* cell wall degradation using [*methyl*-^3^H] choline-labeled pneumococcal cell walls as substrate, following a previously described method (Mosser and Tomasz, 1970). Briefly, 10 μl of enzyme at the appropriate dilution was added to the reaction sample containing 240 μl of PB, 100 mM NaCl, 10 mM DTT, pH 6.8, and 10 μl of radioactively labeled cell walls (≈15000 cpm). After 15 min incubation at 37°C the reaction was stopped by adding 10 μl formaldehyde (37%, v/v) and 10 μl bovine serum albumin (4%, w/v). The pellet was removed by centrifugation (12000 × *g*, 15 min), and the enzymatic activity was quantified by measuring the radioactivity in 200 μl of the supernatant with a liquid scintillation counter (LKB Wallac). One unit of enzymatic activity (U) was defined as the amount of enzyme that catalyzes the hydrolysis (solubilization) of 1 μg of cell wall material in 15 min (Höltje and Tomasz, 1976). Activity assays at different pHs were performed in 20 mM sodium phosphate (pH 5.7–8.0) or 20 mM HCl-Tris buffers (pH 8.0–9.0).

### Bactericidal Assay

Log-phase bacteria were grown to an OD_550_ ≈ 0.3, cooled on ice for 5 min, centrifuged, washed with PB, 100 mM NaCl, pH 6.8, and adjusted to an OD_550_ ≈ 0.6 (10^8^–10^9^ colony forming units (CFUs) per ml) in the same buffer supplemented with 10 mM DTT. Afterward, resuspended bacteria were transferred into plastic tubes and PL3 was added. Controls were always run in parallel, replacing the added enzyme with buffer. Samples were incubated at 37°C for 1 h, the turbidity decrease (OD_550_) was measured, and viable cells were determined using blood agar plates at the end of incubation. For each sample, a 10-fold dilution series was prepared, 10 μl of each dilution was plated, and colonies were counted after overnight incubation at 37°C. Only dilutions with 30–300 colonies were considered, and in assays where the bactericidal effect was high, 100 μl of undiluted suspensions were plated and colonies counted.

### Activity of PL3 in Different Phases of the Growth Curve

*Streptococcus pneumoniae* R6 and P046 strains were incubated until exponential phase of growth and diluted to an OD_550_ of 0.06. Then, cultures were divided in aliquots and PL3 (2.7 μg/ml, final concentration) was added at early exponential phase (OD_550_ ≈ 0.15), late exponential phase (OD_550_ ≈ 0.4), or stationary phase of growth (OD_550_ ≈ 0.7), with or without 10 mM DTT in the medium. Viable cells of treated and untreated samples were counted at 210 min after culture initiation, as explained above.

### Zebrafish Embryo Infection Assay

This study was conducted at The Zebrafish Lab^1^, using wild-type zebrafish embryos that were maintained according to standard protocols (Westerfield, 2007). Briefly, zebrafish embryos were dechorionated at 24 h post fecundation by treatment with pronase (2 mg/ml) for 2 min. At 48 h post fecundation, embryos were individually distributed in 96-well plates and incubated in 50 μl of E3 medium (5 mM NaCl, 0.17 mM KCl, 0.33 mM CaCl_2_ and 0.33 mM MgSO_4_, pH 7) at 28°C in the presence of either alive or heat-killed D39 pneumococcal cells (≈10^8^ CFU/ml) for 8 h. The effect of adding 20 μg of PL3 or 1 mM DTT to uninfected embryos was also tested. Afterward, infected embryos were extensively washed with E3 medium, to remove bacteria, transferred, together with the controls, to new 96-well microtiter plates containing autoclaved E3 fresh medium supplemented with different amounts of PL3 and 1 mM DTT — or the same volume of buffer (controls) — before continuing incubation at 28°C under sterile conditions. Mortality was followed in all samples for 5 days, adding fresh E3 medium without DTT every day. Zebrafish embryos were considered dead when no movement was observed, even if a heartbeat was observed. Opacification of the larvae was always found to follow shortly. Bacterial infection was previously ascertained as the real cause of embryo death by locating fluorescent bacterial signals around the gills (Díez-Martínez et al., 2013).

### Statistical Analysis

All data are representative of results obtained from repeated independent experiments, and each value represents the mean ± standard deviations for three replicates. In the case of the zebrafish embryo assay, the results from four independent experiments were combined to evaluate a total of 256 embryos for controls and for each lysin-treated group. Statistical analysis was performed by using two-tailed Student’s *t-*test (for two groups), whereas analysis of variance (ANOVA) was chosen for multiple comparisons. GraphPad InStat version 3.0 (GraphPad Software, San Diego, CA, USA) was used for statistical analysis.

## Results

### Design and Production of the PL3 Chimera

**FIGURE 1:**
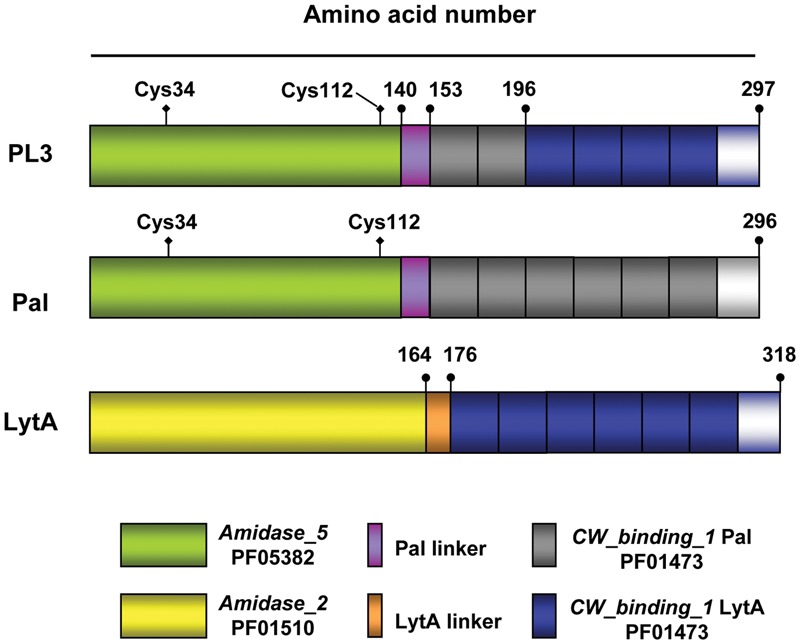
**Schematic representation of the PL3 chimeric NAM-amidase and the parental Pal and LytA murein hydrolases.** Domain and linker origin is depicted by colors; gray and blue rectangles indicate the choline binding repeats comprised in Pal and LytA CBDs, respectively, followed by the C-terminal tail. Numbers show the end of domains and linkers. The position of the two cysteine residues in Pal and PL3 catalytic domains is marked. Pfam entries for *Amidase_2, Amidase_5*, and *CW_binding_1* (choline-binding repeats) families are also shown.

The goal of this work was to construct a new chimeric lysin specifically directed against *S. pneumoniae* and other choline-containing Gram-positive bacteria, with higher activity and stability than the parental enzymes. In this context, we have recently shown how the substitution of the Cpl-7 CBD by a different domain with higher affinity for the substrate resulted in an extremely powerful lysin against pneumococci, i.e., the Cpl-711 chimera (Díez-Martínez et al., 2015). With this aim, we thoroughly analyzed the structural, enzymatic, and bactericidal properties of natural and chimeric lysins from the pneumococcal system, which includes the bacterium and its phages. Thus, we decided to construct the PL3 chimera by combining the catalytic *Amidase_5* domain (PF05382) from Pal, encoded by the bacteriophage Dp-1, and the C-terminal region of the CBD from the major pneumococcal autolysin LytA, which is a member of the *Amidase_2* family (PF01510). The approach was based on the following rationale: (i) both lysins are effective antimicrobials against pneumococci (Jado et al., 2003; Rodríguez-Cerrato et al., 2007); (ii) the catalytic module of Pal is less negatively charged than that of LytA, which might facilitate the lysis from the outside (Low et al., 2011); (iii) saturation of choline-binding sites and choline-induced dimerization, key for lytic activity, occur in LytA at lower ligand concentration than in Pal (Medrano et al., 1996; Varea et al., 2000, 2004); (iv) preservation of Pal overall modular structure in the chimera could be achieved by conserving the linker and the two first choline-binding repeats of Pal (61% sequence identity to those of LytA); and (v) the new chimera will combine the most structurally stable domains of the parental enzymes: the CBD from LytA and the catalytic domain of Pal (Varea et al., 2000, 2004). A comparative scheme of PL3 and the parental Pal and LytA enzymes is shown in **Figure 1** whereas domain and linker charges are given in Supplementary Figure S1 together with amino acid sequence.

**FIGURE 2:**
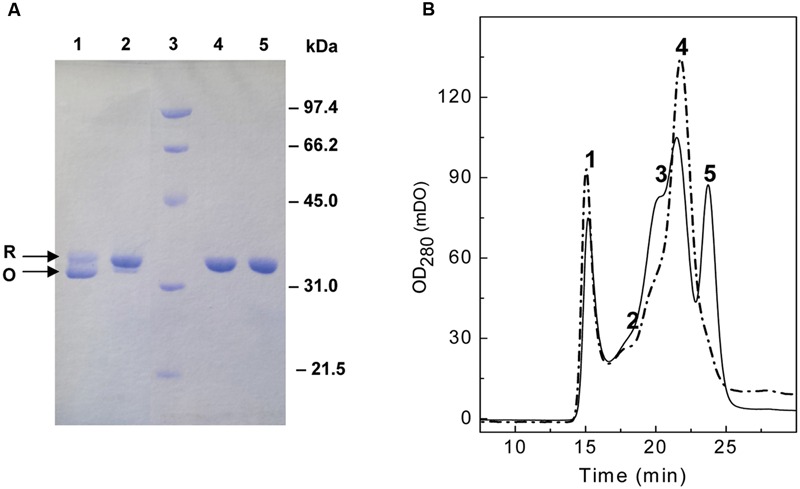
**Analysis of molecular forms of PL3. (A)** Reducing and non-reducing SDS-PAGE profile of PL3. Protein equilibrated in PB or PB with 10 mM DTT was loaded in the gel using sample buffer without (lanes 1 and 2, respectively) or with (lines 4 and 5, respectively) β-mercaptoethanol. Lane 3, standard molecular mass markers. R and O arrows indicate bands corresponding to the reduced and oxidized form of PL3, respectively. **(B)** Size-exclusion fractioning of a PL3 sample, eluted from the DEAE-cellulose column, dialyzed against PB, and pre-equilibrated in a Superose 12 HR/10/30 column with PB, pH 6.8, containing (thick solid trace) or not (gray solid trace) 1 mM β-mercaptoethanol. Injection volume was 100 μl and the flow rate 0.5 ml/min. Large aggregates eluted in peak 1, whereas peak 5 likely corresponds to the oxidized monomer of PL3.

Cloning, overproduction and purification of PL3 were carried out as detailed in the Section “Materials and Methods,” with a yield of ≈110 mg per liter of culture. The protein eluted from the DEAE-cellulose column showed two close bands (R and O) when analyzed under non-reducing conditions by SDS-PAGE. Nevertheless, the faster migrating band (O) disappeared upon pretreatment of the sample with 10 mM DTT (or by adding 1 mM β-mercaptoethanol) to the sample-loading buffer (**Figure 2A**), and the remaining band (R) corresponded with the expected mobility from the theoretical molecular mass of PL3 (34287 Da) and the experimental value measured by MALDI-TOFF mass (34151.3 Da; Met1 is processed). This observation, indicative of an intra-molecular disulphide bridge between the two cysteine residues (Cys34 and Cys112) of PL3 (and Pal) catalytic domain, was consistent with the stimulation of Pal (García et al., 1983) and PL3 (see below) activities by reducing agents. In addition, size-exclusion chromatography of PL3 samples revealed the presence of different association states (**Figure 2B**), including large protein aggregates eluting at the void volume of the column (peak 1), and a likely oxidized form of PL3 (peak 5) observed only when PB used for the protein preequilibration did not contain β-mercaptoethanol. Therefore, PL3 was subjected to a second purification step by size-exclusion chromatography and all the experiments were performed under reducing conditions with protein fractions comprised in peaks 2–4.

Preliminary *in vitro* assays using radioactive pneumococcal cell walls or a suspension of R6 cells as substrates showed that purified PL3 displayed high murolytic and bactericidal activity and, thus, supported our hypothesis that PL3 could be a promising weapon against pneumococci. Therefore, its structural features, choline-binding affinity and structural stability were characterized.

### Characterization of PL3 Structure: Effect of Choline Binding

#### Far- and Near-UV CD Spectra

**FIGURE 3:**
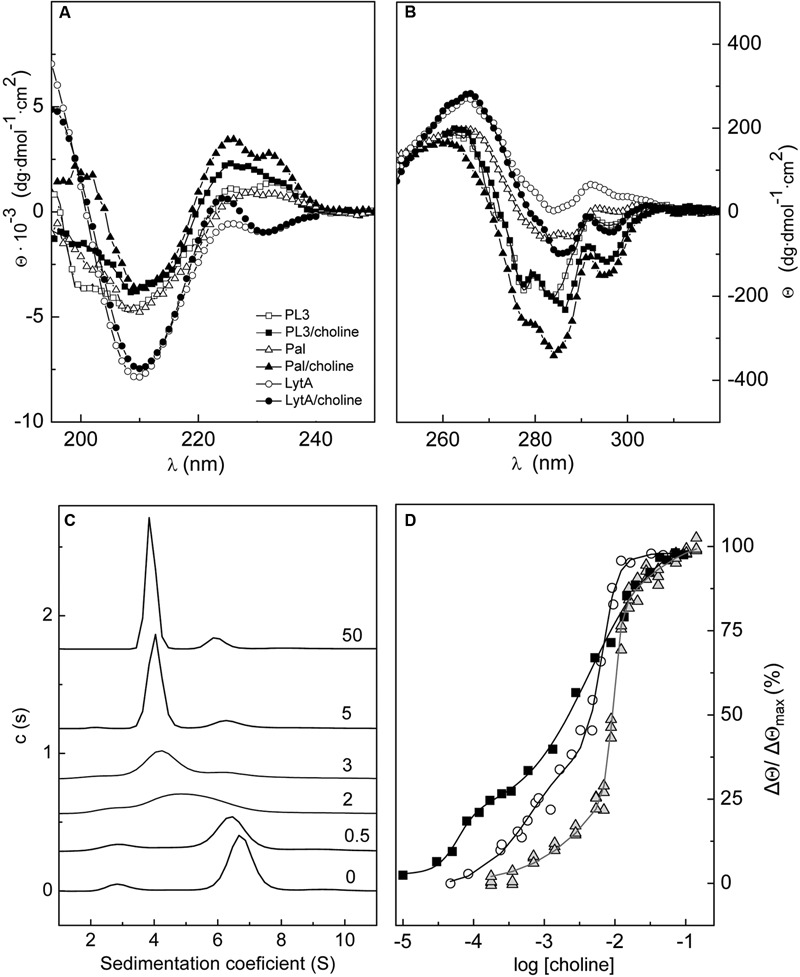
**Effect of choline binding on PL3 structure.** Comparison of the far- **(A)** and near-UV **(B)** CD spectra of PL3, Pal, and LytA in the absence and in the presence of 20 mM choline. **(C)** Effect of choline binding on PL3 association-state distribution. Increase of choline concentration shifts the monomer/dimer/tetramer equilibria of PL3 toward the dimer (*s*_20,w_ = 4.1 S) with the concomitant decrease of monomer (*s*_20,w_ = 3.0) and tetramer (*s*_20,w_ = 6.8 S) populations. Labels indicate the choline concentration (mM). **(D)** CD titration curve of PL3 (squares) generated from the ellipticity changes induced at 295 nm by choline binding. Titration curves of LytA (circles; Varea et al., 2000) and Pal (triangles; Varea et al., 2004) are also depicted. Continuous lines represent the fitting of experimental data as the sum of two sigmoids with the parameters shown in Supplementary Table S1.

The similarity of secondary and tertiary structures among the chimera PL3 and the parental enzymes was analyzed by CD. PL3 and Pal have very similar far-UV spectra (**Figure 3A**), as expected from their identical catalytic domain and linker and the high likeliness (77% sequence identity; 84% similarity) of their CBDs. Main differences found when compared with the LytA spectrum may be attributed to the different folds of their unrelated catalytic domains. Indeed, the two positive maxima displayed by PL3 and Pal spectra at 220–240 nm and the negative band at 200 nm (a shoulder in the Pal spectrum) likely correspond to a fingerprint of the *Amidase_5* domain. Choline binding strongly modified the far-UV spectra of PL3 and Pal; the intensity of the positive peak centered at 225–224 nm was almost doubled, whereas the negative band at 200 nm was highly reduced (the ellipticity became positive for the choline-bound form of Pal). The magnitude of such variations strongly contrasts with the rather local effect of choline binding on the LytA spectrum (the negative maximum at 225 nm became positive upon choline addition). In the near-UV region, largely dominated by the contributions of the CBD aromatic side-chains and sensitive to the tertiary and quaternary structures, the spectrum of unbound PL3 shows features of both parental enzymes, but it reminds the choline-bound spectra of LytA and Pal (**Figure 3B**). The likeness increased upon choline addition, but the intensity of spectrum of the choline-bound chimera was in between those of the parental enzymes.

#### Modulation of PL3 Association States by Choline Binding

Like in other pneumococcal choline-binding proteins carrying a CBD composed of six choline-binding repeats and a C-terminal tail, choline binding regulates PL3 self-association, as depicted in **Figure 3C**. The distribution of sedimentation coefficients (c(*s*^∗^)) of the unbound protein showed a mayor peak with an *s*_20,w_ of 6.8 S corresponding to the tetramer (*M*_w,app_ = 130 kDa), a second peak compatible with the monomer (*s*_20,w_ = 3.0; *M*_w,app_ = 38 kDa) and a minor peak (≤5% total area) at 10.0 S. Choline addition induced PL3 dimerization (*s*_20,w_ = 4.1 S; *M*_w,app_ = 64 kDa) with the subsequent reduction of monomer and tetramer populations. At 5 mM choline and higher, the dimer became the most favored form (≈80% at 0.11 mg/ml PL3) although a small fraction of tetramer (10–12%) was still present. Stabilization of PL3 dimer was concomitant with the saturation of choline higher-affinity sites (see below), a feature shared with LytA, though the most populated form of the unbound autolysin was the dimer (Usobiaga et al., 1996; Varea et al., 2000). In contrast, the predominant species of the unbound and choline-bound forms of Pal were the monomer and the dimer, respectively, which coexisted, however, with lower fractions of higher association states (Varea et al., 2004). In addition, Pal dimerization was enhanced by saturation of the lower affinity sites (Varea et al., 2004). As shown in Supplementary Table S1, the sedimentation coefficients, the frictional coefficient ratios and Stokes radii calculated for a given state are almost identical in the three lysins, considering the higher molecular mass of the LytA monomer, which confirms that they have very similar hydrodynamic shapes.

#### CD Titration of PL3 with Choline

The titration curve of PL3, obtained by representing the relative variation in ellipticity at 295 nm as function of choline concentration (**Figure 3D**), presents two well defined phases, as in Pal and LytA (Medrano et al., 1996; Varea et al., 2004). Saturation of the higher affinity sites required lower choline concentration, compared to the parental enzymes, and correlated with PL3 dimerization. The apparent half-dissociation constants estimated through the description of PL3 titration profile in terms of two sigmoid functions were 60 ± 9 μM and 4.5 ± 0.6 mM for the higher and lower affinity sites, respectively, which are slightly lower than those estimated, with the same approach, for LytA (1.1 and 6.8 mM) and well below those of Pal (8 and 10 mM). In contrast, the cooperativity of choline binding to the lower affinity sites was much higher in the two parental enzymes (Supplementary Table S1).

#### Conformational Stability of PL3

**FIGURE 4:**
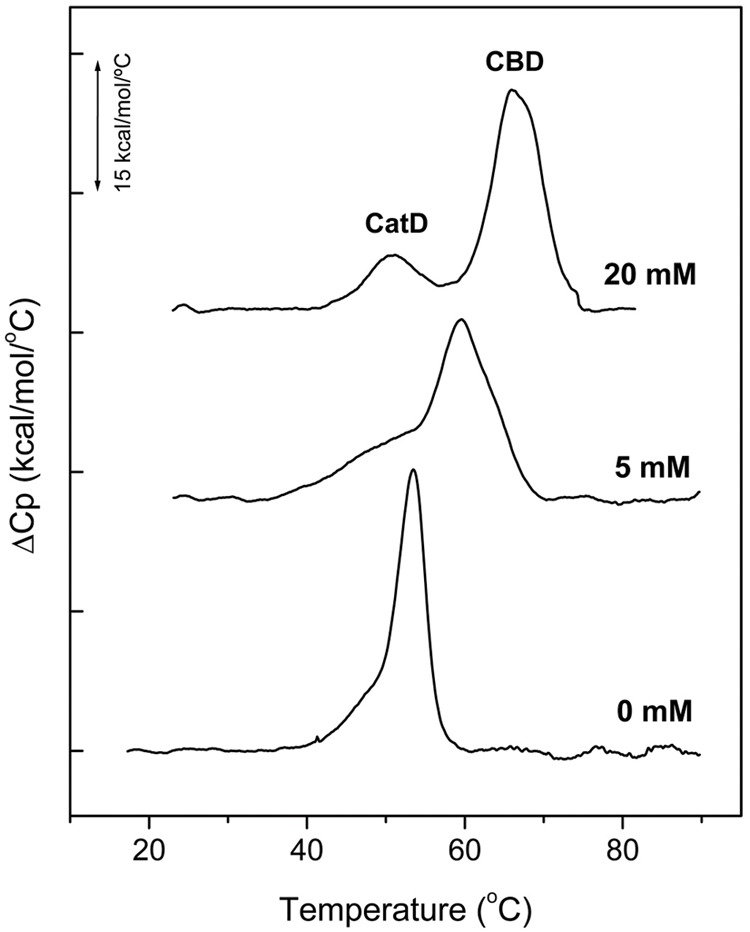
**Thermal denaturation curves of PL3.** DSC profiles of PL3 were measured in the absence and in the presence of choline at ligand concentrations specified in figure labels. Experiments were performed at 0.4 mg/ml PL3 in PB, pH 6.8 (scan rate 60°C/h). The curves were shifted along the *y*-axis for clarity. CatD and CBD indicate the transitions corresponding to the catalytic domain and the cell wall-binding domain, respectively.

Next, we analyzed the conformational stability of PL3 by DSC. The thermal denaturation curves registered in PB at pH 6.8 showed a mayor peak centered at 53.6°C with a shoulder at the lower temperature side (**Figure 4**). The shift of the major peak toward higher temperatures as choline concentration was increased allowed its assignment to the CBD. Moreover, choline-mediated stabilization eliminated the overlapping of the CBD and catalytic domain transitions, the latter becoming fully resolved with a transition temperature of 51°C in the presence of 20 mM choline. Like in Pal and LytA, the CBD stability increased at slightly basic pHs (Usobiaga et al., 1996; Varea et al., 2000, 2004) and was intermediate between those of the parental enzymes. As expected from LytA and Pal stability profiles (Varea et al., 2000, 2004), denaturation of the catalytic domain of PL3 begun around 10°C above that of LytA (Supplementary Figure S2).

### Enzymatic Characteristics of PL3

Once characterized the biophysical properties related with the lytic activity and structural robustness of PL3, we examined its specific activity, very close to those of LytA and Pal (Supplementary Figure S1), and the optimal conditions to either degrade purified pneumococcal cell walls or kill R6 cells. Maximal activity was displayed in PB, 100 mM NaCl, 10 mM DTT, pH 6.8, for the two assays, the effective range of lysis extending from pH 6.5 to 8.0. These results were consistent with the optimal pH values reported for Pal (pH 6.9) and LytA (pH 6.8; Sheehan et al., 1997). The specific activity of PL3, 4 × 10^5^U/mg, was rather similar to that found for the parental proteins, Pal and LytA (Supplementary Figure S3). Remarkably, ≈95% of PL3 murolytic activity was maintained after been stored for 4 weeks at 37°C, and the bacteriolytic activity of the sample was fully preserved when tested against resuspended R6 cells. Moreover, lyophilization of PL3 did not provoke any loss of its murolytic and bacteriolytic capacity (data not shown).

### Bactericidal Activity of PL3

**FIGURE 5:**
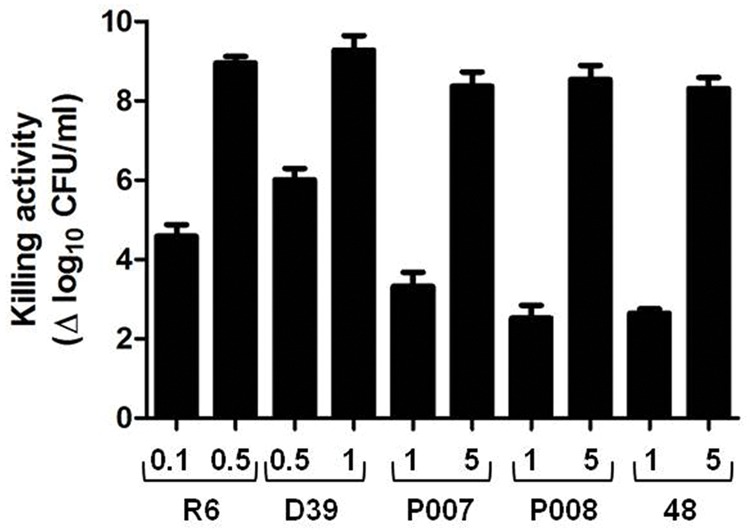
**Bactericidal activity of PL3 against pneumococcal strains.** Exponentially growing cultures were washed, resuspended in PB containing 100 mM NaCl and 10 mM DTT (pH 6.8), and treated with different concentrations of PL3 for 60 min at 37°C. Then, viable cells were measured and killing value was calculated as the decrease of bacterial titers, in log units, compared to PL3-untreated control. Protein concentrations (μg/ml) are indicated in the *x*-axis. Error bars represent standard deviations and differences in activity values are statistically significant (*P <* 0.05).

First, the antibacterial capacity of PL3 was tested against several pneumococcal strains using the protocol described in the Section “Materials and Methods,” which measures the turbidity decrease of bacterial suspensions and the cell survival after 60 min of incubation at 37°C, with and without the lysin. PL3 reduced the viability of all pneumococcal samples tested, including the multiresistant clinical strain 48 (serotype 23F), leading to the practical sterility of the cultures at concentrations of PL3 in the range of 0.5–5 μg/ml (**Figure 5**). In strong contrast with their similar lytic activities on purified cell walls (Supplementary Figure S1B), PL3 kills pneumococci more effectively than Pal and LytA, i.e., PL3 is capable to sterilize an R6 culture at 0.5 μg/ml, whereas Pal and LytA reduced 5 and 7.5 log units the viable cells, respectively, but at 10-fold higher concentration (5 μg/ml; Díez-Martínez et al., 2013). Moreover, at 0.1 μg/ml PL3 was even more lethal than Cpl-1 and Cpl-711 lysozymes against R6 and D39 strains, and as good as Cpl-711 against the multiresistant 48 strain (Díez-Martínez et al., 2015). It is worth noting that the type, thickness and net charge of the different capsular polysaccharides may somewhat modulate the lysin access to the peptidoglycan, as deduced from the higher susceptibility of R6 to the killing action of PL3 compared to that of the corresponding isogenic serotype-2 encapsulated D39 strain. This conclusion is further supported by the lower killing efficiency of PL3 obtained against P007, P008, and 48 strains, belonging to serotypes 3, 4, and 23F, respectively (**Figure 5**).

**FIGURE 6:**
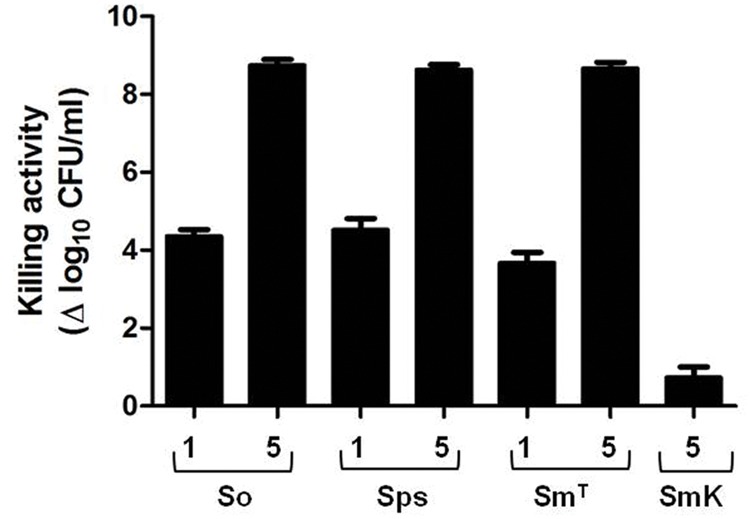
**Bactericidal activity of PL3 against non-pneumococcal species.** The assay was performed as described in **Figure 5**. Numbers on *x*-axis indicate the concentration of PL3, in μg/ml. So, *S. oralis*; Sps, *S. pseudopneumoniae*; Sm^T^, *S. mitis* type strain; SmK, *S. mitis* SK598 strain. Error bars represent standard deviations and differences in activity values are statistically significant (*P <* 0.05).

Since PL3 contains an specific CBD, it was feasible that this chimera could also lyse other Gram-positive pathogens apart from the pneumococcus, provided that they contain choline in the (lipo)teichoic acids. Thus, the bactericidal activity of PL3 was tested on various non-pneumococcal streptococci. As shown in **Figure 6**, PL3 efficiently killed *S. oralis, S. pseudopneumoniae*, and *S. mitis* type strains (harboring choline in their (lipo)teichoic acids), although to eradicate the cells down to the limit of detection it was necessary to add the enzyme at 5 μg/ml. As expected, *S. mitis* SK598 strain, which possesses ethanolamine-containing teichoic acids (Bergström et al., 2003), was refractory to PL3 bacteriolytic action.

### Effect of the Addition of PL3 to Different Phases of the Growth Curve

**FIGURE 7:**
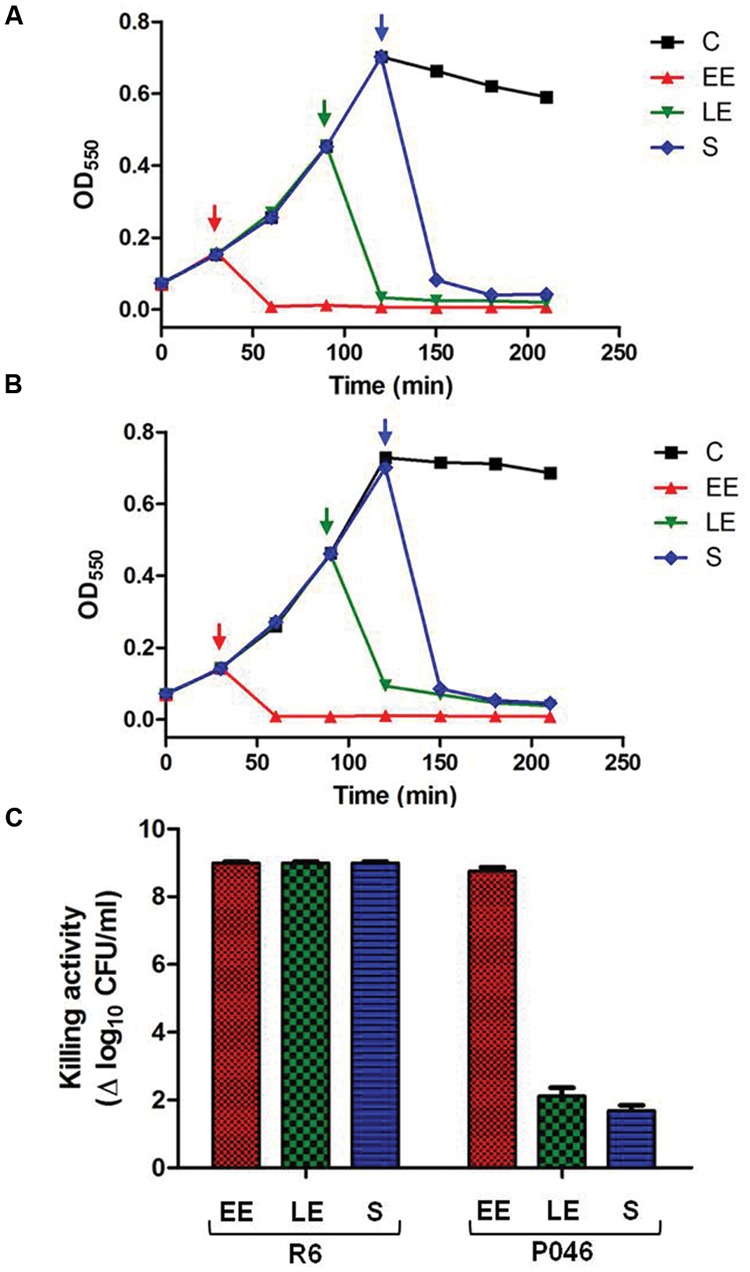
**Effect of PL3 on growth of pneumococcal R6 and P046 cultures. (A,B)** R6 and P046 growing curves where 2.7 μg/ml PL3 were added at different phases of growth (marked by arrows) in the presence of 10 mM DTT. **(C)** Log of killed cells was calculated by comparing CFUs of treated- and PL3-untreated cultures determined 210 min after culture initiation. EE, early exponential phase; LE, late exponential phase; S, stationary phase; C, untreated control. Error bars represent standard deviations and differences in activity values are statistically significant (*P <* 0.05).

After studying the exogenous bacteriolytic action of PL3 on bacterial suspensions, we also investigated its effect along the growing curve of R6 and the isogenic strain P046, a double mutant lacking LytC and LytA autolysins, whose comparison would allow evaluating the influence of endogenous autolysins in PL3-mediated bacteriolysis. Thus, we added the same concentration of PL3 (2.7 μg/ml) to different cultures of R6 or P046 at early exponential (OD_550_ ≈ 0.15), late exponential (OD_550_ ≈ 0.4) and stationary phase of growth (OD_550_ ≈ 0.7) and the turbidity decrease was monitored at 37°C. Viable cells were also determined 3.5 h after culture initiation. The addition of PL3 at any phase of the growth curve produced an immediate and marked OD_550_ decrease, with the concomitant efficient killing of both R6 and P046 cells (**Figures 7A,B**). Notably, the bactericidal effect depended on the strain and the growth phase; whereas PL3 addition sterilized R6 cultures at any point of the growing curve, the number of viable cells for late exponential or stationary P046 cultures was reduced only 2 logs (**Figure 7C**). This difference strongly suggests a synergistic effect between endogenous LytA and LytC autolysins and exogenous PL3 to trigger cell lysis. It is also noteworthy that when the experiment was carried out without DTT in the media, the bacterial population can resume growing again after an initial decrease in the OD_550_ and cell survival was significantly increased, thereby demonstrating that reducing conditions were necessary for the full activity of PL3 (data not shown).

### Bactericidal Activity of PL3 in an Infection Animal Model

**FIGURE 8:**
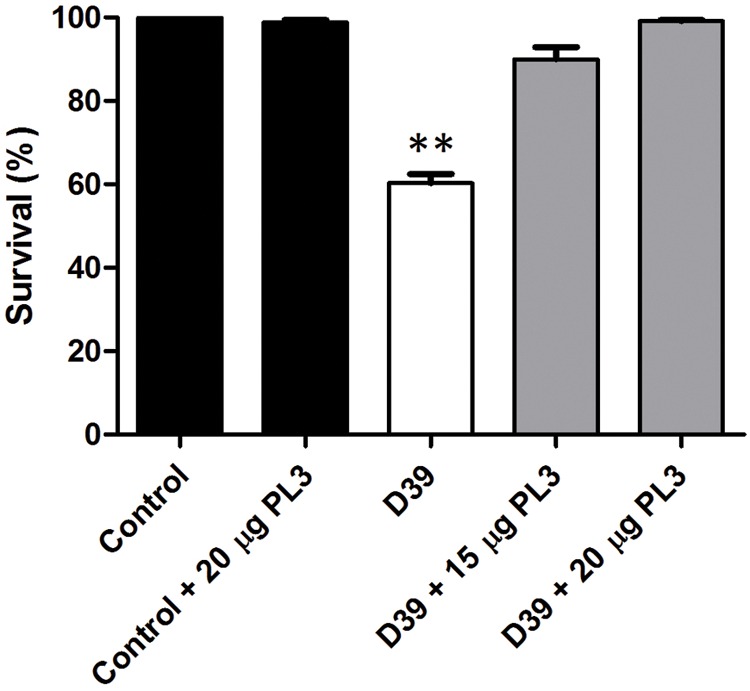
**Rescue of zebrafish embryos from pneumococcal infection by PL3.** Survival of embryos infected with D39 *S. pneumoniae* strain treated or not with single doses of PL3. Data are means from four independent experiments. Error bars represent standard deviation and asterisks mark the results that are statistically significant for the overall comparison of infected or PL3-treated embryos *vs.* the controls (one-way ANOVA with a *post hoc* Dunnett test; ^∗∗^*P* < 0.001).

The experiments described above proved that PL3 efficiently kills pneumococci and relative bacteria *in vitro*. To validate these data *in vivo*, we used the already set up zebrafish embryo model for studying anti-streptococcal compounds (Díez-Martínez et al., 2013). Zebrafish embryos were brought in contact with the pneumococcal strain D39 by immersion in E3 medium containing 1 mM DTT and incubated for 8 h at 28°C, using heat-killed D39 cells (10 min at 65°C) as negative controls. Afterward, embryos were extensively washed with E3 medium and treated with different amounts of PL3, or the corresponding volume of the enzyme buffer, as described in the Section “Materials and Methods.” The mortality rate of infected embryos was around 40%, associated with inflammation of heart and liver, and death occurred at about 48–72 h post-infection. Addition of a single 20-μg dose of PL3 to pneumococcus-infected embryos fully protected them from death, whereas survival dropped to about 90% when treated with 15 μg (**Figure 8**). The level of protection provided by PL3 was higher than those of Cpl-1 or Cpl-7S whose complete protection of embryos was achieved at a dose of 25 μg (Díez-Martínez et al., 2013).

## Discussion

The increased incidence of antibiotic resistance has led to a renewed search for novel antimicrobials. In this context, the use of pathogen-directed antibacterials through the employ of specific lytic peptidoglycan hydrolases appears as an alternative to diminish the rate of antibiotic-resistant pathogens worldwide (Czaplewski et al., 2016). Most lysins reported as effective antibacterials so far have a phage origin, encoded by lytic (virulent) or temperate phages. Besides these “natural” genes as source for lysins, another strategy is to combine catalytic and substrate-binding domains from different origins to construct fusion enzymes with novel bactericidal properties or enhanced activity, solubility or stability (Donovan et al., 2006; Daniel et al., 2010; Díez-Martínez et al., 2015; Yang et al., 2015). Knowledge of the structural and functional properties of selected domains and their compatibility with the envelope structure of the targeted bacterium are key factors to successfully develop tailor-made lysins. Here we describe the design, production and characterization of PL3, a robust chimeric NAM-amidase constructed by substitution of the last four choline binding repeats and the C-terminal tail of the Pal endolysin by those of LytA NAM-amidase, the major pneumococcal autolysin. Remarkably, PL3 bacteriolytic activity from the outside goes beyond those of the parental lysins against all the pneumococcal clones tested, including the 48 strain, which is resistant to penicillin, erythromycin, tetracycline, and quinolones. Depending on the strain, PL3 compares, and even surpasses, the bacteriolytic activity of Cpl-711, the more effective enzybiotic against *S. pneumoniae* so far described (Díez-Martínez et al., 2015). In contrast with some antibiotics, and like other lysins, PL3 also has the advantage of being effective against targeted bacteria at any metabolic state of the culture. In particular, PL3 was able to kill bacteria at the stationary phase of growth when the metabolic machinery, i.e., the level of protein synthesis, was notably reduced. The antimicrobial efficacy of PL3 has been also validated *in vivo* using a zebrafish embryo infection model, where a single dose of 20 μg fully protects against death by infection with pneumococcal D39 strain.

PL3 exhibits also a potent lytic activity against *S. pseudopneumoniae, S. oralis*, and *S*. *mitis* strains that contain choline in the cell wall, which opens up its use to treat the infections caused by these opportunistic pathogens. Namely, *S. pseudopneumoniae* may cause pneumonia, bronchitis and chronic sinusitis with greater resistance than pneumococcus to several antimicrobials (Laurens et al., 2012). *S. mitis* can cause a broad range of infections from caries to invasive diseases like endocarditis, bacteremia, pneumonia, etc., with resistance to common antibiotics (Mitchell, 2011). It is also an emergent causative of blood infections in immunocompromised patients (Shelburne et al., 2014) and has been associated to the toxic shock syndrome with mortality rates above 60% (Tunkel and Sepkowitz, 2002). Finally, *S. oralis* may produce bacterial endocarditis, respiratory distress syndrome and streptococcal shock in immunocompromised individuals (Verhagen et al., 2006). It is also involved in periodontal disease, the most common infection of the human oral cavity (Maeda et al., 2013). In this respect, PL3 is the first lysin reported to effectively kill *S. oralis*.

As expected from previous studies (Usobiaga et al., 1996; Varea et al., 2004), modification of the CBD of Pal leading to the PL3 chimera drastically enhanced choline binding and reduced the concentration required for choline-induced dimerization, two factors shown to be essential for the activity (Varea et al., 2000), without perceptibly affecting the shape of the chimera in relation to the equivalent association state of Pal or LytA. Moreover, saturation of the higher affinity sites occurs in PL3 at lower choline concentration than in LytA, and these features could explain, at least partially, the remarkable superiority of PL3 in eradicating *S. pneumoniae*. These results might also mean that the site located at the interface of the second and third repeat, different in PL3, LytA and Pal, might be central in choline recognition by the CBD. On the other hand, the reduction of PL3 negative net charge in three units with respect to LytA may further increase the bactericidal activity, by facilitating the accessibility of PL3 to the peptidoglycan network through the negatively charged outside of the bacteria (Low et al., 2011; Díez-Martínez et al., 2013). Such effect might also explain why the chimera and the parental lysins display highly different relative activities when tested on bacterial suspensions and very similar on purified pneumococcal cell walls, where substrate fragmentation facilitates the accessibility and cleavage of susceptible bonds.

Domain interchange conferred a remarkable conformational robustness to PL3, evidenced by preservation of around 95% activity when tested on purified pneumococcal cell walls or bacterial suspension after been stored 4 weeks at 37°C. Notably, PL3 can even be lyophilized without any loss of activity when assayed against the two types of substrates. Other interesting finding of this study is the apparent cooperation of bacterial autolysins to the exogenous bacteriolytic action of PL3, which reminds the combined action of LytA and LytC with CbpD in pneumococcal fratricide (Eldholm et al., 2009), and may open new clues about the mechanism of action of lysins as anti-infectives. Specifically, the absence of endogenous LytA and LytC autolysins in pneumococcal P046 strain reduced by around 7 log units the number of killed bacteria in relation with the isogenic R6 strain, when exponential and stationary phase grown cultures were treated with PL3.

Current evolution of the clinical trials is responding to the initial hope of lysins as effective and specific alternative antibacterials to fight most dangerous and multiresistant pathogens. Indeed, the first commercial lysin is already on the market for topical treatment of skin infections provoked by methicillin-resistant *Staphylococcus aureus* (MRSA)^2^. Furthermore, according to the portfolios of several pharmaceutical companies, this example is expected to be followed by other lysins in the near future. The results described in this work show that structure and function-based approach to construct tailor-made lysins by domain shuﬄing from parental proteins is an advantageous alternative and it could be a general method to design a ‘magic bullet’ directed against selected pathogens, provided that in-depth knowledge on the enzymes and substrate characteristics are in hand. In this sense, PL3 could be an appropriate candidate, alone or in combination with other active lysins, for the toolbox to combat multiresistant infections provoked by any pneumococcal strain or closely related pathogens.

## Conclusion

We report here the design, production, and characterization of PL3, a chimeric lysin with a robust bacteriolytic activity *in vivo* and *in vitro* against *S. pneumoniae* and other streptococci bearing choline in the cell wall. Due to its remarkable stability, lyophilization feasibility, and killing efficiency at very low doses, PL3 has a great potential to be used as an effective therapeutic agent against susceptible and multiresistant pathogens.

## Author Contributions

BB, AF-T, MI-B, MM, and PG designed the experiments, performed by BB, AF-T, and MI-B. BB, AF-T, MI-B, MM, and PG discussed the results. MM and PG supervised the study and wrote the manuscript with contributions of BB, AF-T, and MI-B. All authors read, edited, and approved the final manuscript.

## Conflict of Interest Statement

The authors declare that the research was conducted in the absence of any commercial or financial relationships that could be construed as a potential conflict of interest.

## Footnotes

1 http://www.thezebrafishlab.com

2 http://www.micreos.com/
