# Supplementary material for: PL3 Amidase, a Tailor-made Lysin Constructed by Domain Shuﬄing with Potent Killing Activity against Pneumococci and Related Species
Source: Front Microbiol. 2016 Jul 28;7:1156. doi: 10.3389/fmicb.2016.01156 (PMC4963390; doi:10.3389/fmicb.2016.01156)
Supplement: Supplementary file 1 [file Data_Sheet_1.PDF]

A

10	20	30	40	50
MGVDIEKGVA	WMQARKGRVS	YSMDFRDGPD	SYDCSSSMYY	ALRSAGASSA
60	70	80	90	100
GWAVNTEYMH	AWLIENGYEL	ISENAPWDAK	RGDIFIWGRK	GASAGAGGHT
110	120	130	140	150
GMFIDSDNII	HCNYAYDGIS	VNDHDERWYY	AGQPYYYVYR	LTNANAQPAE
160	170	180	190	200
KKLGWQKDAT	GFWYARANGT	YPKDEFEYIE	ENKSWFYFDD	QGYMLADRWR
210	220	230	240	250
KHTDGNWYWF	DNSGEMATGW	KKIADKWYYF	NEEGTMKTGW	VKYKDTWYYL
260	270	280	290	297
DAKEGAMVSN	AFIQSADGTG	WYYLKPDGTL	ADKPEFTVEP	DGLITVK

B

Net charge				Protein	Specific Activity (U/mg) ×10^{-5}	Choline IC50 (mM)	Origin
Z_{CatD}	Z_{linker}	Z_{CBD}	Z_{total}				
-4.7	1.0	-7.8	-11.5	PL3	4.0 ± 0.9[a]	9	Synthetic
-4.7	1.0	-6.8	-10.5	Pal	1.2 ± 0.1[b]	2	Lytic phage
-8.0	-1.9	-4.7	-14.6	LytA	2.5 ± 0.2[b]	24	*S. pneumoniae* R6

Figure S1. Amino acid sequence and characteristics the PL3 chimera. (A) The one letter amino acid code is highlighted according to the color code used in **Figure 1** to identify the origin of the catalytic domain (CatD; Pal, green), linker (Pal, violet)**,** choline binding repeats of the CBD (Pal, grey; LytA, blue) and the C-terminal tail of the latter. The last six amino acids of the second repeat (GYMLAD) are common to the two parental enzymes. Cysteine residues are underlined. (B) Net charge, specific activity, half choline inhibitory concentration (IC50) and origin of PL3 and the parental lysins. Z_{CatD}, Z_{link}, Z_{CBD}, and Z_{total} correspond, respectively, to the net charge of the CatD, the linker, the CBD and the total net charge. [a]Measured at 37°C in PB, pH 6.8, 10 mM DTT. [b]Data from Sheehan et al. (1997).

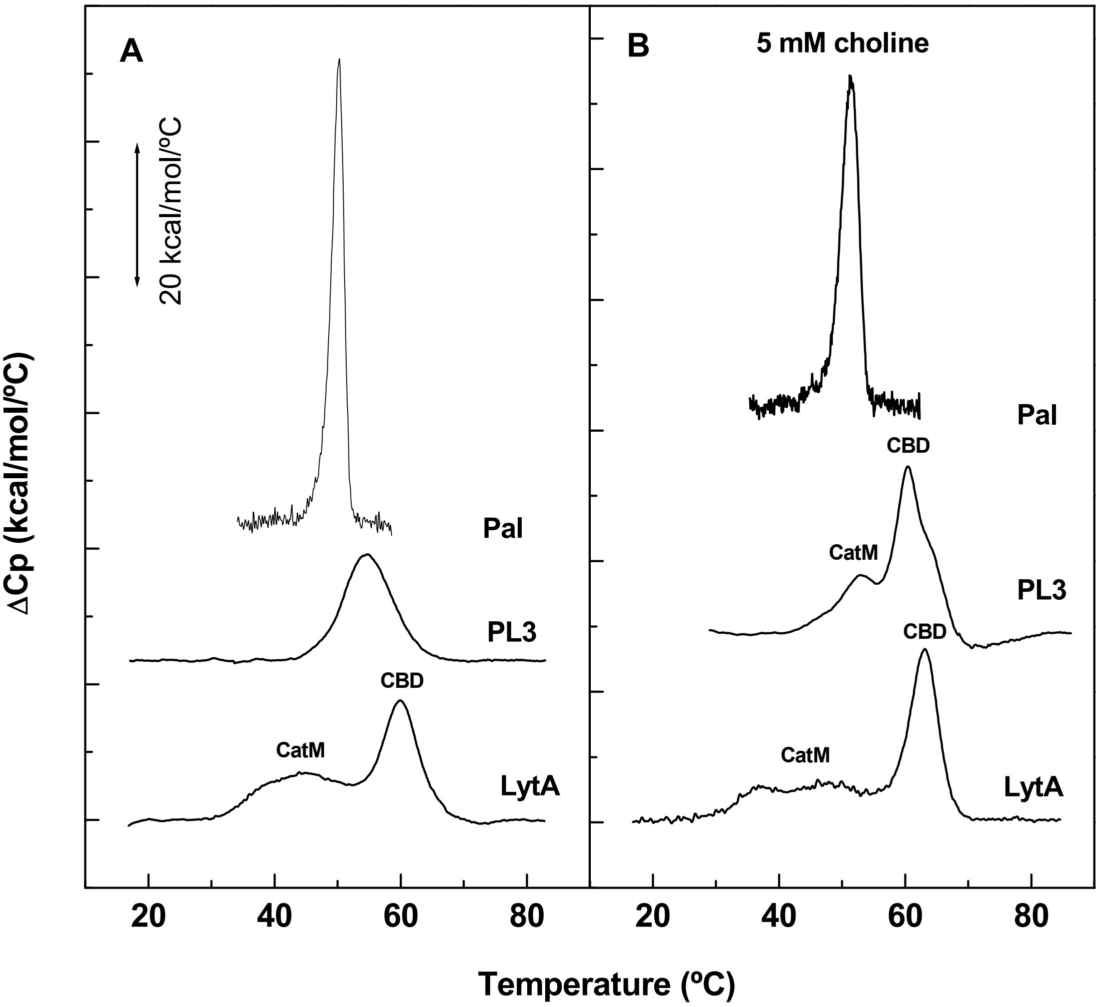

SUPPLEMENTARY FIGURE S2 I Thermal stability of PL3, Pal and LytA. Comparison of PL3 and parental murein hydrolases thermal stability in the absence **(A)** and in the presence **(B)** of 5 mM choline. Thermograms registered in PB, pH 8.5 (PL3) or pH 8.0 (Pal[a] and LytA[b]); curves were shifted along the y-axis for clarity (CatD and CBD indicated the position of transitions assigned to the catalytic domain and the choline-binding domain, respectively, when appear resolved in the thermograms. [a]Data from Varea et al. (2004). [b]Data from Varea et al. (2000).

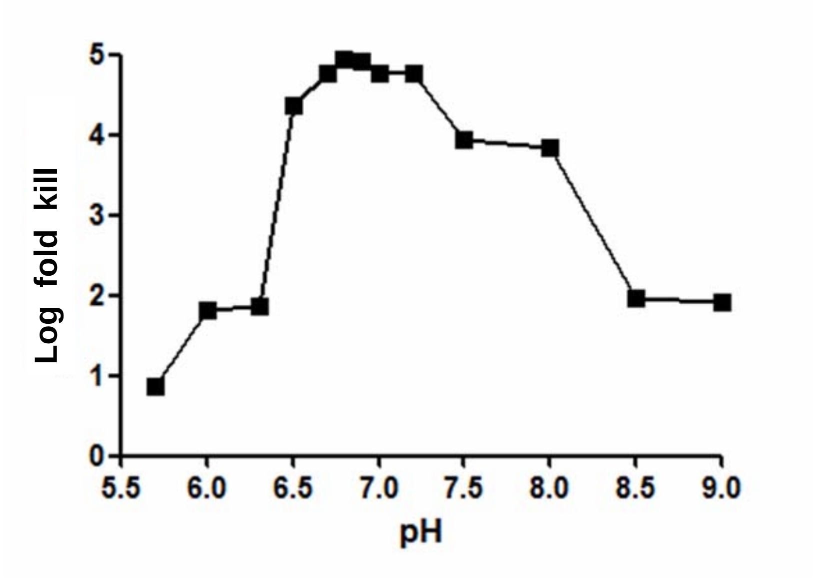

Supplementary Figure S3 I pH dependence of PL3 bactericidal activity. Exponentially growing R6 cells were washed and suspended in PB containing 100 mM NaCl and 10 mM DTT, at different pHs, in the presence of 0.1 µg/ml of PL3. Incubation continued for 60 min at 37°C. Then, viable cells were counted and killing activity was calculated as the difference between bacterial titers (CFU), in log units, of PL3-treated and untreated controls.

SUPPLEMENTARY TABLE S1 I Parameters of major forms of PL3, LytA and Pal

	PL3			LytA	Pal	
	monomer	dimer	tetramer[a]	dimer	monomer	dimer
**Molecular mass** (kDa)	34,151	68,302	136,604	73,088	34,453	68,906
**Sedimentation velocity**						
$s_{20,w}$ (S)	3.0	4.1	6.8	4.1	3. 3	4.2
f/f_0	1.25	1.51	1.38	1.56	1.27	1.52
R_s (Å)	27.2	41.5	47.9	43.9	27.8	41.0
**Choline titration**[b]						
K_1^{app}	60 ± 9 (µM)			1.1 ± 0.5 (mM)	8 ± 3 (mM)	
p_1^{app}	2.1 ± 0.7			1.0 ± 0.2	0.8 ± 0.2	
K_2^{app}	4.5 ± 0.6 (mM)			6.8 ± 0.4 (mM)	9.7 ± 0.5 (mM)	
p_2^{app}	0.9 ± 0.1			5 ± 2	7 ± 2	

[a]Parameters calculated for the tetramer without choline. [b]K_1^{app} *and* K_2^{app} are the apparent half dissociation constants for the higher and lower affinity sites, respectively, estimated by fitting two sigmoid functions to the experimental data. p_1^{app} and p_2^{app} describe the sigmoid slope.
